# Matrix metalloproteinase-9 predicts pulmonary status declines in α_1_-antitrypsin deficiency

**DOI:** 10.1186/1465-9921-12-35

**Published:** 2011-03-23

**Authors:** Theodore A Omachi, Mark D Eisner, Alexis Rames, Lada Markovtsova, Paul D Blanc

**Affiliations:** 1 Division of Pulmonary, Critical Care, and Sleep Medicine, Department of Medicine, University of California San Francisco, San Francisco, CA, USA; 2 Cardiovascular Research Institute, University of California San Francisco, San Francisco, CA, USA; 3 Genentech Inc., South San Francisco, CA, USA; 4 Roche Pharmaceuticals, Basel, Switzerland; 5 Roche Pharmaceuticals, South San Francisco, CA, USA; 6 Division of Occupational and Environmental Medicine, Department of Medicine, University of California, San Francisco, San Francisco, CA, USA

## Abstract

**Background:**

Matrix metalloproteinase-9 (MMP-9) may be important in the progression of emphysema, but there have been few longitudinal clinical studies of MMP-9 including pulmonary status and COPD exacerbation outcomes.

**Methods:**

We utilized data from the placebo arm (n = 126) of a clinical trial of patients with alpha_1_-antitrypsin deficiency (AATD) and emphysema to examine the links between plasma MMP-9 levels, pulmonary status, and COPD exacerbations over a one year observation period. Pulmonary function, computed tomography lung density, incremental shuttle walk test (ISWT), and COPD exacerbations were assessed at regular intervals over 12 months. Prospective analyses used generalized estimating equations to incorporate repeated longitudinal measurements of MMP-9 and all endpoints, controlling for age, gender, race-ethnicity, leukocyte count, and tobacco history. A secondary analysis also incorporated highly-sensitive C-reactive protein levels in predictive models.

**Results:**

At baseline, higher plasma MMP-9 levels were cross-sectionally associated with lower FEV_1 _(p = 0.03), FVC (p < 0.001), carbon monoxide transfer factor (p = 0.03), resting oxygen saturation (p = 0.02), and ISWT distance walked (p = 0.02) but were not associated with radiographic lung density or total lung capacity (TLC). In longitudinal analyses, MMP-9 predicted a further decline in transfer factor (p = 0.04) and oxygen saturation (p < 0.001). MMP-9 also predicted worsening lung density (p = 0.003), increasing TLC (p = 0.02), and more frequent COPD exacerbations over follow-up (p = 0.003). Controlling additionally for hs-CRP levels did not substantively change the longitudinal associations between MMP-9 and these outcomes.

**Conclusions:**

Increased plasma MMP-9 levels generally predicted pulmonary status declines, including worsening transfer factor and lung density as well as greater COPD exacerbations in AATD-associated emphysema.

## Introduction

Chronic obstructive pulmonary disease (COPD) is a leading cause of morbidity and mortality worldwide, but predictors of progression remain elusive [1]. Alpha_1_-antitrypsin inhibits neurophil elastase, and the discovery of alpha_1_-antitrypsin deficiency (AATD) and its association with emphysema and COPD helped to establish the concept that an imbalance between proteases and antiproteases, exacerbated by exposure to cigarette smoke, can play a key role in the development of disease, at least in a subset of COPD [2]. Evolving evidence suggests that a variety of proteinases, acting on diverse substrates of the extracellular matrix, play an important role in pulmonary parenchymal destruction in COPD [3,4]. The study of these proteinases has been complicated by the diversity of COPD phenotypes, which include components of emphysema and chronic bronchitis, as well as the common co-morbid cardiovascular and other systemic diseases associated with COPD, all of which potentially confound the analyses of biomarker-disease associations [1,5]. Because AATD-associated emphysema represents a more homogenous phenotype of COPD, with a more rapid decline in pulmonary status, generally at a younger age with fewer comorbidities, it offers a potential model for understanding COPD pathogenesis with respect to proteinases beyond those directly inhibited by alpha_1_-antitrypsin [2,6].

Matrix metalloproteinase-9 (MMP-9) is one such proteinase that has received considerable attention in COPD[7-18]. Clinical studies have suggested that, among the various biomarkers potentially associated with lung disease, MMP-9 may be particularly important, although there have been few longitudinal studies of MMP-9 and few pulmonary status outcomes examined [7-10]. Of the 143 serologic biomarkers examined by Pinto-Plata and colleagues, MMP-9 was the most highly correlated with the BODE Index (cross-sectionally) and with COPD exacerbations (longitudinally over 12 months) [7]. Additionally, the ratio of biomarker in COPD subjects to that in health controls was higher for MMP-9 than for any of the other biomarkers examined [7]. Higashimoto and colleagues examined multiple biomarkers among 96 COPD patients, finding that only MMP-9 and C-reactive protein were statistically significantly associated with declines in FEV_1 _over the following 12 months [8]. Neither these nor other longitudinal studies of MMP-9 have investigated pulmonary status measured by transfer factor for carbon monoxide (TLco), radiographically-quantified lung density, or exercise capacity. Studies to date have furthermore not incorporated multiple longitudinal measurements of MMP-9. Finally, clinical studies thus far have not reported on the longitudinal associations between MMP-9 and outcomes within the context of AATD-associated emphysema, a phenotype of COPD that could be particularly relevant to understanding the role of MMP-9 in the natural history of COPD pathogenesis.

Utilizing data from the placebo arm of a clinical trial, we tested the hypotheses that plasma MMP-9 levels are cross-sectionally and prospectively associated with pulmonary status declines and with COPD exacerbations among emphysema patients with AATD.

## Methods

### Overview

We analyzed data from the placebo arm of the Retinoids in Emphysema Patients on Alpha_1_-Anitrypsin International Registry (REPAIR) trial, which has previously been described in detail [19]. This was a randomized double-blind trial that investigated the potential for a retinoid agonist to slow the progression of emphysema in AATD patients. Using measurements collected as part of this clinical trial, we investigated the cross-sectional and longitudinal association between plasma MMP-9 and multiple pulmonary endpoints, including COPD exacerbation frequency. For example, in longitudinal analyses, our goal was to determine the anticipated change in pulmonary status that would be predicted by higher levels of plasma MMP-9 in a given individual represented by this cohort.

### Participants

For the REPAIR trial, Pi Z and Pi Null genotype subjects with emphysema were recruited from 10 alpha_1_-antitrypsin registries in 10 countries. 126 subjects with AATD-associated emphysema were randomized to the placebo arm of the trial and provided plasma samples sufficient for analysis of MMP-9. Inclusion criteria and exclusion have previously been described [19]. Notable inclusion criteria were: (1) no alpha_1_-antitrypsin augmentation therapy for at least 28 days prior to enrolment, (2) TLco < 70% and a post-bronchodilator FEV_1 _≤ 80% of the predicted values for sex, age, and height; (3) no use of systemic corticosteroids within 28 days prior to enrolment; and (4) ability to perform an incremental shuttle walk test (ISWT) without supplemental oxygen. Subjects were excluded if (1) they had experienced >3 COPD exacerbations in the year prior to enrolment or (2) were current tobacco smokers or had smoked tobacco in the 6 months prior to enrolment. The REPAIR trial was approved by relevant local ethics review committees and was conducted in accordance with the Declaration of Helsinki and Good Clinical Practice guidelines. All patients provided written informed consent.

### Biomarker Analysis

We focused our analysis on MMP-9 because of *a priori *evidence of its importance in COPD [7-12,20], although various biomarkers were measured as part of the REPAIR trial. As a secondary analysis, however, we also included plasma levels of highly-sensitive C-reactive protein (hs-CRP) in longitudinal multivariable analyses.

For protein biomarker analyses, venous whole blood samples were collected into standard vacutainer tubes containing EDTA as an anticoagulant. After centrifugation for 10 minutes at 1,800 × g at 4°C, samples were kept frozen at -70°C until measurement. All samples for any given subject over the study period were run simultaneously after the subject completed the 52 week trial period. Plasma MMP-9 and hs-CRP levels were quantified at baseline, 3 months and 6 month by Pathway Diagnostics (Malibu, CA). MMP-9 was analyzed using Searchlight^® ^MMP-9 assays (Pierce, Rockford, IL). This assay consists of sandwich enzyme-linked immunosorbent assay for the quantitative measurement of total MMP-9 (the combination of pro-form and active form MMP-9). The enzyme-substrate reaction produces a chemiluminescent signal detected with a cooled Charge-Coupled Device camera (Pierce). The representative sample range for this MMP-9 assay, obtained from duplicate analysis of 20 human subjects without known history of active disease, is 4.2 ng/ml to 120 ng/ml. Although serum has sometimes been used in the analysis of MMP-9 [7-9], analysis of plasma MMP-9 has been shown to provide superior accuracy [21]. Plasma hs-CRP levels were determined by use of the Immulite^® ^High Sensitivity CRP test kit (Siemens Healthcare Diagnostics, Deerfield, IL). For both MMP-9 and hs-CRP quantification, each sample was assayed twice and the mean value of the two measurements was used. The mean intra-assay coefficient of variation (CV) was <15% for both assays. As a covariate in all multivariable analyses, we also included leukocyte count since this could be related to MMP-9 levels; leukocyte count was available from standard complete blood count (CBC) analyzed in a central laboratory.

**Figure 1:**
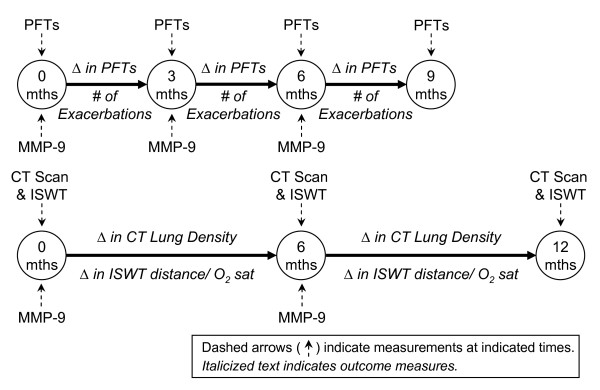
**Timeline of repeated measures models for longitudinal analyses**. Several repeated measures models were used to assess the longitudinal association of MMP-9 with the number (#) of subsequent COPD exacerbations or the subsequent changes (Δ) in various pulmonary status measures. The schedule of assessments for different pulmonary status measures varied in the REPAIR trial, necessitating different modeling time periods.

Baseline biomarker results were used for cross-sectional analyses. For longitudinal analyses, we examined the predictive association of biomarker results from the baseline, 3 month, and 6 month time-points with the frequency of subsequent COPD exacerbations and subsequent changes in pulmonary status (lung function, exercise capacity and radiographic lung density). As discussed below, the schedule of assessments for different pulmonary status measures varied in the REPAIR trial, necessitating different modeling time periods in longitudinal repeated measures analyses (Figure 1).

### Study Endpoints

#### 1. Pulmonary Function Testing (PFT)

PFT measurements, including TLco, post-bronchodilator FEV_1 _and forced vital capacity (FVC), and total lung capacity (TLC) were assessed at baseline, 3 months, 6 months, and 9 months, according to European Respiratory Society (ERS) guidelines [22,23]. Based on spirometry results, patients were staged by Global Initiative for Chronic Obstructive Lung Disease (GOLD) criteria [24]. TLC was measured by body box plethysmography when possible, but helium dilution was used at study sites that did not have access to a body plethysmograph or if subjects were unable to perform box plethysmography (25% of subjects in this analysis had TLC measured by helium dilution). The study protocol required that the same method for assessing TLC had to be used with each subject in longitudinal follow-up. MMP-9 levels were not statistically significantly different between sites that used body plethysmograph and helium dilution techniques at baseline, 3 months, or 6 months (p-values for difference, based on Wilcoxon rank-sum test were 0.24, 0.52, and 0.94 respectively).

We assessed the cross-sectional baseline correlation of plasma MMP-9 with FEV_1_, FVC, FEV_1_/FVC ratio, TLC, and TLco (unadjusted for lung volume or hemoglobin). We furthermore determined the prospective association of (1) MMP-9 at baseline with the changes in PFT measurements (FEV_1_, FVC, FEV_1_/FVC ratio, TLC and TLco) between baseline and 3 months, (2) MMP-9 at 3 months with the changes in PFT measurements between 3 months and 6 months, and (3) MMP-9 at 6 months with PFT changes between 6 months and 9 months.

#### 2. Exercise Testing and Oxygen Saturation

Incremental shuttle walk tests (ISWT) were performed at baseline, 6 months, and 12 months using standard protocols [25]. The primary outcome measure for the ISWT is total distance walked. Resting room air oxygen saturation by pulse oximetry was obtained prior to ISWT and used separately for analysis. The baseline correlations of MMP-9 with both ISWT distance and resting oxygen saturation were determined. We furthermore determined the prospective association between (1) baseline MMP-9 and both change in ISWT distance and change in oxygen saturation between baseline and 6 months, and (2) MMP-9 at 6 months with change in ISWT distance and oxygen saturation between 6 months and 12 months.

#### 3. Computed Tomography (CT) Lung Densitometry

The proteolytic tissue destruction that is pathognomonic of emphysema should directly cause a reduction of lung density. Lung density changes can be measured using CT scanning, and CT lung densitometry is thought to be the most sensitive and specific measure of emphysema in vivo [26-31]. Specifically, CT scanning has been validated as a measure of emphysema by studies that have shown good correlation with pathology [32,33], exercise capacity [34], health status [34] and lung function [30,35]. Furthermore, CT densitometry is a better predictor of mortality than lung function in AATD-associated emphysema [36]. For this reason, change in CT-measured lung density was chosen as the primary end-point to examine the efficacy of the study drug in the REPAIR trial [19].

CT scans were performed at baseline, 6 months, and 12 months. For the purposes of volume adjustment, two CT scans were performed at each visit, one scan during a breath-hold at TLC followed immediately by a second scan at approximately functional residual capacity (FRC) [37]. Lung density was assessed as the value in Hounsfield Units (HU) at which 15% of the lung voxels have a lower density (15^th ^percentile method) [26]. We assessed both the cross-sectional baseline correlation of MMP-9 with volume-adjusted lung density as well as the prospective association of MMP-9 with changes in volume-adjusted lung density. Specifically, for prospective analyses, we determined (1) the association of MMP-9 at baseline with the change in lung density between baseline and 6 months and (2) the association of MMP-9 at 6 months with the change in lung density between 6 months and 12 months.

#### 4. Acute Exacerbations of COPD

Patients in the REPAIR trail were seen in clinic at least every 4 weeks (more often if they developed new complaints) to assess for adverse events. As part of this ascertainment, any new prescriptions for antibiotics or systemic corticosteroids for respiratory reasons were recorded, along with the dates of administration. Following precedent and the recommendation of working groups and disease experts, a COPD exacerbation was defined as a prescription of systemic corticosteroids or antibiotics for worsening respiratory status [38-40]. As advocated by Burge and Wedzicha, we classified various prescriptions as a single episode when the interval between adjacent contacts was <14 days [38].

### Statistical Analysis

#### Primary Analyses

Statistical analysis used Stata/SE software (version 9.2, College Station, TX). This was an exploratory post-hoc analysis, and because of the risk of Type II error, we did not adjust for multiple comparisons resulting from either the multiple outcomes examined or the fact that other data were potentially available for analyses [41]. Our *a priori *hypotheses focused on the potential associations of MMP-9 with COPD progression and outcomes.

All multivariable analyses of MMP-9 controlled for age, gender, race-ethnicity, leukocyte count, and tobacco pack-year history. Each of these factors was considered to be a potential confounder in the relationship between MMP-9 levels and poor outcomes. For example, leukocytes may release MMP-9 and, potentially, could independently be a predictor of future COPD exacerbations. Therefore, leukocyte count obtained simultaneous to the point in time of MMP-9 measurements was included as a covariate in cross-sectional and longitudinal analyses. In our analysis of TLC, we also controlled for method of TLC assessment (body box plethysmography *vs*. helium dilution).

Baseline cross-sectional associations of MMP-9 with study end-points were assessed using multivariable linear regression, with the aforementioned study end-points as the dependent variable and MMP-9 and covariates as independent variables.

Because prospective analyses involved multiple assessments of MMP-9 and outcomes for each subject over time, we used a repeated measures analysis that incorporated all periods of follow-up data simultaneously. Specifically, we used generalized estimating equations to take into account the fact that a given subject contributed more than one observation to the analysis [42-45]. All outcomes other than COPD exacerbations were modelled as changes in a measurement from one period to the next, and such changes were well-approximated by a normal distribution, which was incorporated into our modelling.

For the endpoint of COPD exacerbations, the number of COPD exacerbations within each of the 3 month observation periods followed a Poisson distribution, and our generalized estimating equations thus incorporated such a distribution to model not simply the presence or absence of a COPD exacerbation but the number of exacerbations in any given 3 month time-frame.

As a descriptive analysis, we also evaluated (1) the longitudinal change in MMP-9 levels as well as (2) the change in each outcome measure over the study period irrespective of MMP-9 levels. We examined the latter to gauge the magnitude of the overall changes in each measurement, to better place into context the changes associated with MMP-9 levels. We used the paired t-test to examine whether these changes were different than zero. For pulmonary function measurements, which were done every 3 months over a 9 month period, we presented the overall change as an annualized rate to make it comparable to the other measurements.

#### Secondary Analyses

C-reactive protein (CRP) has been studied extensively in COPD and has been shown to predict declines in lung function as well as COPD outcomes, such as risk of hospitalization and death from COPD [46,47]. CRP is an acute-phase reactant and is associated with systemic as well as lung inflammation in COPD [46]. As such, whether or not MMP-9 may predict COPD progression or outcomes after controlling for CRP levels may be of interest both to (1) those wishing to understand whether MMP-9 may add incremental predictive value above and beyond CRP as well as (2) those wishing to know if any associations between MMP-9 and pulmonary outcomes persist after controlling for non-specific inflammation using a well-validated measure. In this analysis, therefore, we controlled additionally for hs-CRP levels in our multivariable models. Furthermore, we utilized the Spearman rank test to examine correlations between hs-CRP and MMP-9 levels at baseline, 3 months, and 6 months.

To provide insight into whether MMP-9 might add incremental predictive value above and beyond degree of disease severity, we conducted a secondary analysis, based on our longitudinal multivariable models, in which we controlled for either FEV_1 _or FEV_1_/FVC ratio in addition to the covariates used in our core analyses (age, gender, race-ethnicity, leukocyte count, and tobacco pack-year history). For this analysis, we utilized FEV_1 _and FEV_1_/FVC ratio results that were obtained simultaneous to the point in time at which MMP-9 was measured (i.e., at the beginning of each longitudinal period of follow-up).

Because a COPD exacerbation may theoretically affect the level of plasma MMP-9 and independently be associated with subsequent COPD exacerbations, we performed another secondary analysis in which we controlled for prior COPD exacerbations. For example, in this secondary analysis, the modelling of COPD exacerbations between 3 and 6 months controlled for whether subjects had had a COPD exacerbation between baseline and 3 months. This analysis was not able to control for the presence or absence of COPD exacerbations prior to baseline because such data were not available, although by exclusion criteria patients could not have been treated with systemic corticosteroids in the 28 days prior to baseline nor had >3 exacerbations in the year prior to enrolment.

## Results

### Subject Characteristics

The mean subject age at baseline was 53.8 (SD = 8.5 years), 27% of subjects were female, and all subjects were Caucasian (Table 1). The vast majority of subjects (89%) had a tobacco smoking history, with an average 19 pack-year history (SD = 12.4) among ever smokers. Per study exclusion criteria, there were no known current smokers or recent ex-smokers in the group. The mean FEV_1 _% predicted was 46.5% (SD = 16.8%), and the mean FEV_1_/FVC ratio was 0.38 (SD = 0.11). Additional baseline subject characteristics are shown in Table 1.

**Table 1:** Baseline characteristics of 126 subjects with AATD-associated emphysema

	Mean ± SD or N (%)
Age, years	53.8 ± 8.5

Female	34 (27%)

Caucasian race	126 (100%)

Tobacco Status	
Never Smoker	14 (11%)
Former Smoker*	112 (89%)

Pack-Years (among former smokers)	19.0 ± 12.4

Body-Mass Index	25.2 ± 4.3

FEV_1_,% predicted	46.5% ± 16.8%

FEV_1_/FVC	0.38 ± 0.11

Total Lung Capacity, % predicted	149% ± 45%

GOLD Stage	
1	21 (17%)
2	56 (44%)
3	47 (37%)
4	2 (2%)

Transfer Factor (TLco), mmol/min/kpa	4.9 ± 1.6

Transfer Factor, % predicted	48% ± 15%

CT Densitometry	
Adjusted Lung Density^†^	-956.2 ± 16.0

Resting Oxygen Saturation (room air)	93.5% ± 2.8%

Incremental Shuttle Walk Test	
Distance Walked, meters	406 ± 203

MMP-9 level (ng mL^-1^)	
Median (25^th ^to 75^th ^Interquartile Range)	28.3 (17.8 - 55.4)

*5 subjects (4%) had quit smoking 6-12 months prior to baseline. The remainder of former smokers had quit smoking >12 months prior to baseline. There were no current smokers by study exclusion criteria.

^†^Measured in Hounsfield Units at the 15^th ^percentile.

### Baseline Association of MMP-9 with Pulmonary Status

After controlling for sociodemographic factors, tobacco history, and leukocyte count, MMP-9 at baseline was associated with multiple deficits in pulmonary status (all changes stated per 25^th^-75^th ^percentile interquartile range [IQR) increment in MMP-9). As shown in Table 2, these deficits were: lower FEV_1 _(-32 ml; 95% confidence interval [CI] -61 to -4; p = 0.03), lower FVC (-105 ml; 95% CI -160 to -52; p < 0.001), lower TLco (-0.1 mmol/min/kpa; 95% CI -0.2 to -0.01; p = 0.03), lower resting oxygen saturation (-0.2%; 95% CI -0.3% to -0.03%; p = 0.02), and less distance walked on the ISWT (-12 meters; 95% CI -22 to -2; p = 0.02). MMP-9 was not statistically associated at baseline with lung density, FEV_1_/FVC ratio, or TLC (Table 2).

**Table 2:** Baseline cross-sectional association of plasma MMP-9 with pulmonary status among 126 alpha_1_-antitrypsin deficient subjects with emphysema

	Δ (95% CI)*	P-value
Spirometry		
FEV_1_, ml	-32 (-61 to -4)	0.03
FVC, ml	-105 (-160 to -52)	<0.001
FEV_1_/FVC ratio	+0.2% (-0.3% to +0.7%)	0.47

Total Lung Capacity, ml	-64 (-154 to +26)	0.16

Transfer Factor (TLco), mmol/min/kpa	-0.1 (-0.2 to -0.01)	0.03

CT densitometry		
Adjusted Lung Density^†^	-0.24 (-1.1 to +0.6)	0.58

Resting O_2 _saturation (room air)	-0.2% (-0.3% to -0.03%)	0.02

Incremental Shuttle Walk Test		
Distance Walked, meters	-12 (-22 to -2)	0.02

All analyses controlled for age, gender, race-ethnicity, leukocyte count, and tobacco pack-year history. Analysis of total lung capacity also controlled for method of lung capacity assessment (box plethysmography *vs *helium dilution).

*Change (Δ) associated cross-sectionally with a 25^th ^- 75^th ^percentile interquartile range higher level of MMP-9.

^†^Measured in Hounsfield Units at the 15^th ^percentile.

### Longitudinal Changes in Pulmonary Status and MMP-9

**Figure 2:**
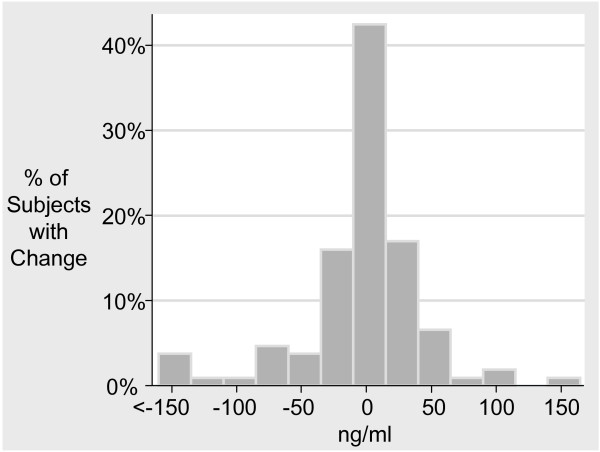
**Change in MMP-9 levels by subject from baseline to 6 months**.

Between baseline and 6 months, MMP-9 declined on average by 13.2 ng/ml (95% CI -33.6 to +7.1; p = 0.20); changes in MMP-9 by subject are presented in Figure 2. Without taking into account MMP-9 levels, we evaluated the change in pulmonary status measurements over the study period and present these results on an annualized (12 month) basis. TLco declined on average by 0.2 mmol/min/kpa (95% CI -0.34 to -0.08; p = 0.002) while lung density declined on average by 1.8 HU (95% CI -2.4 to -1.1; p < 0.001). FEV_1 _declined by 36 ml (95% CI -67 to -5; p = 0.025) and FEV_1_/FVC ratio declined by 1% (95% CI -2% to -0.02%; p = 0.046). The changes in other outcome measurements were not statistically significant (Table 3).

**Table 3:** Change in pulmonary status measurements over the entire study period

	Annualized change in measurements over study period	
	
	Mean (95% CI)*	P-value*
Spirometry^†^		
FEV_1_, ml	-36 (-67 to -5)	0.025
FVC, ml	-6 (-113 to +102)	0.92
FEV_1_/FVC ratio	-1% (-2% to -0.02%)	0.046
Total Lung Capacity, ml^†^	-48 (-152 to +56)	0.36
Transfer Factor (TLco), mmol/min/kpa^†^	-0.2 (-0.34 to -0.08)	0.002
CT Densitometry		
Adjusted Lung Density^‡^	-1.8 (-2.4 to -1.1)	<0.001
Resting Oxygenation Saturation (room air)	0.2% (-0.3% to +0.6%)	0.43
Incremental Shuttle Walk Test		
Distance Walked, meters	-8 (-23 to +6)	0.26

*95% confidence interval (CI) and P-value were calculated using the paired t-test, which determined the 95% CI of the observed changes in each measurement over the study period and tested whether these changes were different than 0.

^†^Change in pulmonary function testing (PFT) measurements, which for the purposes of this study were measured over a 9 month period, are presented after extrapolation to an annualized (12 month) basis.

^‡^Measured in Hounsfield Units at the 15^th ^percentile.

As shown in Table 4, an interquartile range higher level of MMP-9 predicted a 0.1 mmol/min/kpa annual decline in TLco (95% CI -0.2 to -0.004; p = 0.04), a 0.6 HU annual decline in radiographic lung density (95% CI -1.1 to -0.2; p = 0.003), and a 0.5% absolute annual decline in oxygen saturation (95% CI -0.7% to -0.3%; p < 0.001). MMP-9 was not statistically associated with a fall in the distance walked on the ISWT (-6 meters; 95% CI -15 to +2; p = 0.16). Higher levels of MMP-9 were associated with a an increase in FVC (+72 ml; 95% CI +6 to +138; p = 0.03) and an increase in TLC (+69 ml; 95% CI +10 to +126; p = 0.02). MMP-9 manifested a borderline association with a decline in the FEV_1_/FVC ratio (-0.6%; 95% CI -1.3% to +0.01%; p = 0.053), but, in contrast to the general pattern, an increase in absolute FEV_1 _(+14 ml; 95% CI -4 to +33; p = 0.13).

**Table 4:** MMP-9 as a longitudinal predictor of respiratory outcomes: lung function, lung density, functional status, and COPD exacerbations

Outcome Measures	Annualized Δ (95% CI)*	P-value
Spirometry		
FEV_1_, ml	+14 (-4 to +33)	0.13
FVC, ml	+72 (+6 to +138)	0.03
FEV_1_/FVC ratio	-0.6% (-1.3% to -0.1%)	0.053

Total Lung Capacity, ml	+69 (+10 to +126)	0.02

Transfer Factor (TLco), mmol/min/kpa	-0.1 (-0.2 to -0.004)	0.04

CT Densitometry		
Adjusted Lung Density^†^	-0.6 (-1.1 to -0.2)	0.003

Resting Oxygenation Saturation (room air)	-0.5% (-0.7% to -0.3%)	<0.001

Incremental Shuttle Walk Test Distance		
Distance Walked, meters	-6 (-15 to +2)	0.16

Acute Exacerbations of COPD, number	+0.27 (+0.10 to +0.45)	0.003

All analyses controlled for age, gender, race-ethnicity, leukocyte count, and tobacco pack-year history. Analysis of total lung capacity also controlled for method of lung capacity assessment (box plethysmography *vs *helium dilution).

*Annualized longitudinal change (Δ) predicted by a 25^th ^- 75^th ^percentile interquartile range higher level of MMP-9.

^†^Measured in Hounsfield Units at the 15^th ^percentile.

### COPD Exacerbations

In each of the three 3 month periods, the number of COPD exacerbations followed an approximately Poisson distribution with 20% to 22% of subjects experiencing at least 1 COPD exacerbation in each period. Over all 3 time periods, 42% of subjects experienced at least one COPD exacerbation. An interquartile range higher level of MMP-9 predicted an annualized average of 0.27 additional COPD exacerbations (95% CI 0.10 to 0.45; p = 0.003) (Table 4).

### Secondary Analyses

As shown in Table 5, controlling additionally for hs-CRP levels in multivariable longitudinal analyses did not meaningfully alter the relationship between MMP-9 and the outcomes studied. Furthermore, hs-CRP levels were not predictive of changes in pulmonary status, except in the case of resting oxygen saturation (p = 0.001), nor was CRP predictive of COPD exacerbations. CRP manifested a borderline association with declines in ISWT distance (p = 0.07). In Spearman's rank test, MMP-9 levels were not statistically associated with hs-CRP levels at baseline (rho = 0.13; p = 0.14), 3 months (rho = 0.004; p = 0.97), or 6 months (rho = 0.01; p = 0.92).

**Table 5:** Secondary Analysis: The addition of hs-CRP into longitudinal models of respiratory outcomes did not substantively change the predictive relationship between MMP-9 and the outcomes examined.

Outcome Measures	MMP-9 Annualized Δ* (95% CI) p-value	hs-CRP Annualized Δ* (95% CI) p-value
Spirometry		
FEV_1_, ml	+15 (-4 to +34) p = 0.12	-25 (-73 to +22) p = 0.29
FVC, ml	+72 (+6 to +139) p = 0.03	-28 (-192 to +136) p = 0.73
FEV_1_/FVC ratio	-0.6%(-1.3% to +0.01%) p = 0.053	-1.3%(-2.9% to +0.3%) p = 0.11

Total Lung Capacity, ml	+69 (+10 to +127) p = 0.02	+17 (-156 to +190) p = 0.85

Transfer Factor (TLco), mmol/min/kpa	-0.1 (-0.2 to -0.002) p = 0.047	-0.07 (-0.4 to -0.2) p = 0.63

CT Densitometry		
Adjusted Lung Density^†^	-0.6 (-1.0 to -0.2) p = 0.006	+0.7 (-0.3 to +1.7) p = 0.16

Resting Oxygenation Saturation (room air)	-0.4%(-0.6% to -0.2%) p < 0.001	-0.9% (-1.4 to -0.4) p = 0.001

Incremental Shuttle Walk Test Distance, meters	-6 (-14 to +3) p = 0.20	-20 (-42 to +1) p = 0.07

Acute Exacerbations of COPD, number	+0.26 (+0.08 to +0.45) p = 0.006	+0.16 (-0.6 to +0.9) p = 0.67

All analyses included age, gender, race-ethnicity, leukocyte count, tobacco pack-year history, MMP-9 levels, and hs-CRP levels as independent variables in a multivariable model. Analysis of total lung capacity also controlled for method of lung capacity assessment (box plethysmography *vs *helium dilution).

*Annualized longitudinal change (Δ) predicted by a 25-75^th ^interquartile range higher level of MMP-9 or hs-CRP, respectively.

^†^Measured in Hounsfield Units at the 15^th ^percentile.

**Figure 3:**
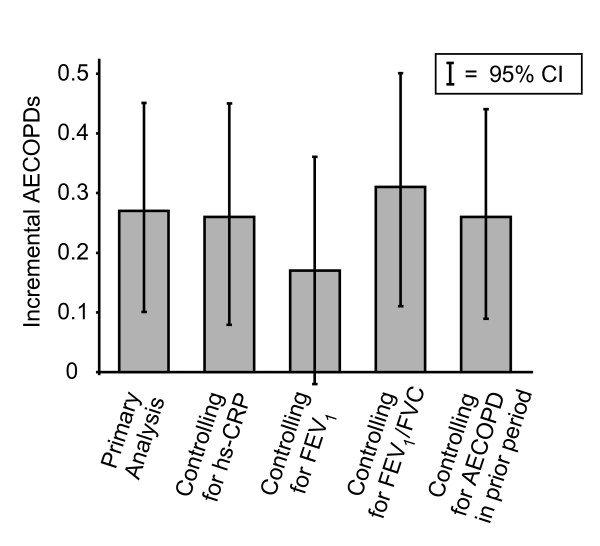
**Primary and secondary analyses of incremental annual acute exacerbations of COPD (AECOPDs) predicted per interquartile higher level of MMP-9**. The primary analysis controls for age, gender, race-ethnicity, leukocyte count, and tobacco pack-years. Secondary analyses control for the same covariates as those used in primary analyses *and* one of the following: hs-CRP, FEV_1_, FEV_1_/FVC, or COPD exacerbation in the prior period.

Controlling additionally for either FEV_1 _or FEV_1_/FVC ratio did not substantively alter the predictive relationship between MMP-9 and the outcomes and various measures of disease progression presented in Table 4, with the possible exception of COPD exacerbations; when controlling for FEV_1_, the number of COPD exacerbations predicted by a interquartile higher level of MMP-9 declined from 0.27 (95% CI 0.10 to 0.45; p = 0.003) to 0.17 (95% CI -0.02 to 0.36; p = 0.08) (Figure 3). Estimated effect sizes or levels of statistical significance for the other outcome measures presented in Table 4 were not substantively changed when controlling additionally for FEV_1 _or FEV_1_/FVC ratio (results not shown).

Because COPD exacerbations may be a risk factor for subsequent COPD exacerbations and may also theoretically result in higher MMP-9 levels, we performed a further analysis in which we controlled for prior COPD exacerbation, in addition to potential confounders used in the core analyses. In that analysis, MMP-9 level retained its role as a predictor of subsequent COPD exacerbations (0.26 additional COPD exacerbations; 95% CI 0.09 to 0.44; p = 0.004). Results of various secondary analyses for the outcome of COPD exacerbations are presented in Figure 3.

## Discussion

This study provides evidence that MMP-9 is a biomarker that predicts COPD progression. Our insights were facilitated by repeated longitudinal measurements of both MMP-9 and a variety of measures of pulmonary status. Moreover, we carried out this longitudinal analysis of MMP-9 in a relatively large cohort of AATD-associated emphysema, a well defined phenotype that offers a particularly valuable model of COPD progression [2].

We found that higher plasma levels of MMP-9 were associated with poorer pulmonary status at baseline and that MMP-9 was longitudinally associated with certain decrements in pulmonary status and worse health outcomes. Of note, however, this was not universally the case for all of the pulmonary status measures we studied, suggesting that the role of MMP-9 is likely to be complex and, in particular, time-dependent. Nonetheless, the *longitudinal *associations between higher levels of MMP-9 and declines in TLco and lung density are both findings that support higher MMP-9 levels being a marker for pulmonary parenchymal destruction. Thus, our longitudinal findings with respect to lung density and TLco suggest that, in AATD-associated emphysema, further study of a direct involvement of MMP-9 in disease progression is warranted.

The magnitude of the prospective associations between MMP-9 and pulmonary status measurements was, in many cases, modest from a clinical perspective, but the time period examined was short relative to the multi-year timeframe over which emphysema progresses. Indeed, the longitudinal changes observed in the various pulmonary status measures, irrespective of MMP-9 levels, were themselves small. That many findings associated with MMP-9 were statistically significant, despite the relatively short period of follow-up, is thus all the more noteworthy.

Although MMP-9 was cross-sectionally associated with lower FEV_1_, its longitudinal association with obstructive ventilatory defects was ambiguous, with higher MMP-9 levels being associated prospectively with a lower FEV_1_/FVC while associated with a higher FEV_1_, albeit of borderline statistical significance (p = 0.13). Research on MMP-9's association with airway pathology is limited [14], but this finding does stand in contrast to that of Hashimoto and colleagues [8]. On the other hand, Vignola and colleagues, in their cross-sectional study, also found a positive correlation between MMP-9 and higher FEV_1 _[48]. One theoretical explanation for a paradoxical association with decreased FEV_1_/FVC ratio but increased FEV_1 _would be that destruction of pulmonary parenchyma, and its associated airway tethering, may potentially result in greater reductions in longitudinal than radial airway traction and thus work to increase small airway caliber [49]. It is also possible that this finding was simply a chance observation, especially given that the longitudinal association with increased FEV_1 _did not meet criteria for statistical significance. Moreover, the annualized decline in FEV_1 _of 36 ml in this cohort is substantially lower than the 54 ml annual decline that has previously been observed in AATD-associated emphysema [50]. This finding magnifies the inherent limitations of studying FEV_1_, an endpoint that can be problem-ridden in airways disease because of its variability and effort dependence. Indeed, this was part of the impetus for choosing lung density, rather than FEV_1_, as the primary outcome in the REPAIR trial [19].

The fact that the longitudinal associations observed with respect to MMP-9 persisted even after controlling for both leukocyte count and hs-CRP levels argues that these predictive observations are not merely reflective of MMP-9 as a non-specific inflammatory marker. Indeed, MMP-9 was poorly correlated with hs-CRP levels. Further, hs-CRP was itself a relatively poor predictor of changes in pulmonary status and did not predict COPD exacerbations. CRP has shown some variable ability to predict COPD progression and outcomes, although this has generally been examined in non-AATD cohorts which included active smokers, who were excluded here [46,47,51]. Thus, the positive associations found with respect to MMP-9 are more remarkable in the context of an absence of consistently positive findings associated with CRP levels.

Our study was not designed to establish a causative role of MMP-9 in antitrypsin deficient emphysema progression. Evidence from other studies suggest that MMP-9 degrades basement membrane type IV collagen, insoluble elastin, and other matrix proteins important to the function and structural integrity of the lung parenchyma [14-16]. MMP-9 has also been shown, through amino terminal processing, to potentiate interleukin-8 chemotactic activity for neutrophils ten-fold, providing an additional inflammatory pathway for lung damage and COPD exacerbations [18]. Nonetheless, we cannot exclude the possibility that our observations could be attributable to a confounding correlation between MMP-9 and other factors that contribute to COPD exacerbations or the progression of emphysema. For example, MMP-9 is released primarily by neutrophils and macrophages; these leukocytes could by themselves be responsible for some of our observations, independent of MMP-9. Our analysis controlled for leukocyte count, and the presumed primary mechanism of action for MMP-9 is biologically coherent in playing a causative role in some of the effects we observed, but this study supports only the conclusion that MMP-9 is a biomarker that predicts disease progression but not necessarily that MMP-9 plays an etiologic role.

There are additional limitations of our research beyond those alluded to above. Despite the advantages in studying AATD-associated emphysema, our findings may not be generalizable to COPD in the absence of AATD and, indeed, the AATD registry from which patients were recruited for this study represents only a subset of AATD. Further, this study was also conducted in subjects who had been non-smokers for at least 6 months, and it is unclear whether current tobacco smoking might alter the relationship between MMP-9 and pulmonary outcomes. The assay used to quantify MMP-9 measured total MMP-9 concentration rather than MMP-9 enzymatic activity as measured by bioassay. While measurement of enzymatic activity may have been preferable in providing greater ability to find associations [52], reduced accuracy from relying on total MMP-9 would tend to diminish our ability to demonstrate statistically significant associations, but does not argue against the validity of associations that we did observe. Finally, assessments of MMP-9 and pulmonary status and outcomes were not performed on an ideal schedule and frequency. For instance, some measurements, such as PFTs, were available every 3 months while others, such as lung density, were available every 6 months. The lack of an MMP-9 measurement at 9 months also places constraints on our analysis. In the best of all possible worlds, an MMP-9 assessment far more frequently than every 3 months (e.g., every two weeks) might have provided data clarifying MMP-9 fluctuations in relation to disease outcomes, in particular COPD exacerbations. Nonetheless, the repeated longitudinal measurements in this study, although imperfect, are a key strength of the analysis.

## Conclusions

Among patients with AATD-associated emphysema, our data show that higher plasma MMP-9 levels were generally associated, both cross-sectionally and longitudinally, with poorer and declining pulmonary status, across multiple endpoints. Overall, this study supports prior research indicating MMP-9 may be a valuable biomarker for the progression of emphysema.

## Competing interests

The authors declare that they have no competing interests.

## Authors' contributions

TAO contributed to the study design, data analysis, and developed the first draft of the manuscript. MDE contributed to the study design, data analysis, and manuscript preparation. AR contributed to the study design and manuscript preparation. LM contributed to the data analysis and manuscript preparation. PDB contributed to the study design, data analysis, and manuscript preparation. All authors read and approved the manuscript.
